# Effects of the Pre-Consolidated Materials Manufacturing Method on the Mechanical Properties of Pultruded Thermoplastic Composites

**DOI:** 10.3390/polym14112246

**Published:** 2022-05-31

**Authors:** Alexander Vedernikov, Kirill Minchenkov, Sergey Gusev, Artem Sulimov, Ping Zhou, Chenggao Li, Guijun Xian, Iskander Akhatov, Alexander Safonov

**Affiliations:** 1 Center for Materials Technologies, Skolkovo Institute of Science and Technology, 30/1 Bolshoi Boulevard, 121205 Moscow, Russia; kirill.minchenkov@skoltech.ru (K.M.); s.gusev@skoltech.ru (S.G.); a.sulimov@skoltech.ru (A.S.); i.akhatov@skoltech.ru (I.A.); 2 Key Lab of Structures Dynamic Behavior and Control, Harbin Institute of Technology, 73 Huanghe Road, Nangang District, Harbin 150090, China; gszhouping@foxmail.com (P.Z.); lichenggao02@126.com (C.L.); gjxian@hit.edu.cn (G.X.); 3 Key Lab of Smart Prevention and Mitigation of Civil Engineering Disasters of the Ministry of Industry and Information Technology, Harbin Institute of Technology, 73 Huanghe Road, Nangang District, Harbin 150090, China; 4 School of Civil Engineering, Harbin Institute of Technology, 73 Huanghe Road, Nangang District, Harbin 150090, China

**Keywords:** fiber-reinforced materials, thermoplastic composite, thermoplastic pultrusion, thermoplastic resin, thermoplastic prepregs

## Abstract

The choice of a manufacturing process, raw materials, and process parameters affects the quality of produced pre-consolidated tapes used in thermoplastic pultrusion. In this study, we used two types of pre-consolidated GF/PP tapes—commercially available (ApATeCh-Tape Company, Moscow, Russia) and inhouse-made tapes produced from commingled yarns (Jushi Holdings Inc., Boca Raton, FL, USA)—to produce pultruded thermoplastic Ø 6 mm bars and 75 mm × 3.5 mm flat laminates. Flat laminates produced from inhouse-made pre-consolidated tapes demonstrated higher flexural, tensile, and apparent interlaminar shear strength compared to laminates produced from commercial pre-consolidated tapes by as much as 106%, 6.4%, and 27.6%, respectively. Differences in pre-consolidated tape manufacturing methods determine the differences in glass fiber impregnation and, thus, differences in the mechanical properties of corresponding pultruded composites. The use of commingled yarns (consisting of matrix and glass fibers properly intermingled over the whole length of prepreg material) makes it possible to achieve a more uniform impregnation of inhouse-made pre-consolidated tapes and to prevent formation of un-impregnated regions and matrix cracks within the center portion of the fiber bundles, which were observed in the case of commercial pre-consolidated tapes. The proposed method of producing pre-consolidated tapes made it possible to obtain pultruded composite laminates with larger cross sections than their counterparts described in the literature, featuring better mechanical properties compared to those produced from commercial pre-consolidated tapes.

## 1. Introduction

Recent decades have been marked by the wide adoption of fiber-reinforced polymer (FRP) materials [1]. Depending on the polymer matrix used, two types of FRP composites can be distinguished—thermoset and thermoplastic ones [2]. Compared to their thermoset counterparts, thermoplastic FRP composites offer numerous advantages, such as higher impact toughness [3], recyclability [4], lower environmental impact [5], higher production speed [6], weldability [7], and bendability [8]. Pultrusion is claimed to be the most efficient process for the production of thermoplastic FRP composites with constant cross sections [9]. Composite profiles of various cross sections (flat laminates, hollow box, I-, U-, C-, L-, and T-shape profiles) are widely used in various sectors of industry—transportation [10], aerospace [11,12], marine construction [13], civil engineering, and architecture [14,15,16,17]. Moreover, due to their high strength-to-weight ratio and excellent corrosion resistance, FRP composite bars have gained engineers’ attention as a possible substitute for steel bars used in reinforced concrete structures [18,19]. In addition, their nonmagnetic, noncorrosive, and nonconductive characteristics make FRP composite bars a good alternative to traditional central strength members of steel used in power transmission cables [20]. 

In traditional thermoset pultrusion, the reinforcement material in the form of unidirectional rovings, mats, fabrics, or veils is impregnated with a polymer matrix. Then, the impregnated pack is fed into the heated die where composite polymerization takes place [21]. Further on, the system of puller units pulls the solidified composite profile to the cut-off saw where the profile is cut to desired lengths [21]. The low viscosity of thermoset resins, which is 2–3 orders of magnitude lower than that of thermoplastic ones [9], makes it possible to carry out the impregnation process in a bath filled with polymer matrix. However, this impregnation method cannot be applied in thermoplastic pultrusion, thus necessitating the use of towpregs [22,23], commingled yarns [24,25], and pre-consolidated tapes [26,27] as pre-impregnated materials. In pre-consolidated tapes (PCTs) the reinforcement material is already impregnated, making PCTs the material of choice among other pre-impregnated materials described earlier [22]. Properties of pultruded thermoplastic profiles depend on the quality of the PCTs, which, in turn, is determined by the manufacturing process and process parameters [28]. Although the relationships between source materials [22,29,30], process parameters [23,26,31,32], and characteristics of pultruded thermoplastic composites have been extensively investigated, the influence of the PCT manufacturing process on the mechanical characteristics of pultruded thermoplastic composites still needs further investigation. Moreover, despite the fact that the morphology analysis of thermoset [33] and thermoplastic [34,35,36] composites has been performed previously, the influence of the choice of source materials on the morphology of pultruded thermoplastic composites requires deeper analysis.

This study investigated the mechanical properties and morphology of pultruded thermoplastic glass fiber/polypropylene (GF/PP) bars (Ø 6 mm) and flat laminates (75 mm × 3.5 mm) produced from two different types of PCTs manufactured by different processes. In the study we used commercially available PCTs and their inhouse-made counterparts based on glass fiber/polypropylene (GF/PP) commingled yarns. Pultruded composite elements were tested in tension, flexure, and interlaminar shear. The study also included optical microscopy analysis of inhouse-made and commercial PCTs and of pultruded composites based thereupon. The results showed that the proposed method for PCT manufacture, based on commingled yarns, had a considerable influence on the mechanical properties of the corresponding pultruded thermoplastic composite.

## 2. Materials and Methods

### 2.1. Manufacturing of PCTs

Two types of PCTs were used to manufacture thermoplastic composites: commercial (ApATeCh-Tape Company, Russia) and inhouse-made tapes. Commercial thermoplastic PCTs consist of 2400 TEX unidirectional glass fiber rovings and the Moplen RP388U polypropylene matrix. The volume fraction of reinforcement constituted 38.6%. The tapes had a width of 4.83 mm and a thickness of 0.66 mm. The commercial thermoplastic PCTs were produced by injecting the polypropylene resin into the die block where the glass fiber reinforcement was fed into. Due to confidentiality reasons, the manufacturer chose not to disclose the design of the die block used in the production of the commercial PCTs and the respective information on process parameters.

Inhouse-made thermoplastic PCTs were produced at the Laboratory of Composite Materials and Structures of the Center for Materials Technologies (Skolkovo Institute of Science and Technology, Moscow, Russia), using a Plastron FLD 35 (Zhangjiagang Friend Machinery Co., Ltd., Zhangjiagang, China) extrusion machine. In order to produce inhouse-made thermoplastic PCTs, we used the modified die block with corrugated cavity walls and glass fiber/polypropylene (GF/PP) commingled yarns (Jushi Holdings Inc., USA).

### 2.2. Pultrusion Setup

In order to produce thermoplastic composites, we used the Pultrex Px500-6T (Pultrex, Lawford, UK) pultrusion machine at the Laboratory of Composite Materials and Structures of the Center for Materials Technologies (Skolkovo Institute of Science and Technology, Moscow, Russia). Two types of pultruded thermoplastic GF/PP profiles were produced in the course of the study—Ø 6 mm bars and 75 mm × 3.5 mm flat laminates.

#### 2.2.1. Manufacturing of Thermoplastic Bars

**Figure 1:**
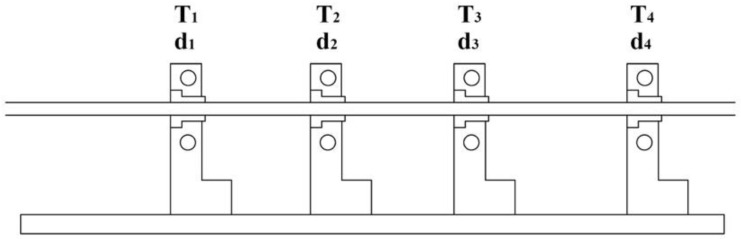
Position of die blocks for the pultrusion of thermoplastic GF/PP bars of Ø 6 mm, manufactured from inhouse-made PCTs.

In order to produce thermoplastic GF/PP bars Ø 6 mm we used 3 heated die blocks and one cooling die installed along the pultrusion direction. The die block dimensions were 60 mm × 103.5 mm. The die orifice diameters were d_1_ = 7.1 mm, d_2_ = 6.5 mm, and d_3_ = 6.2 mm, respectively. The cooling die had an orifice of d_4_ = 6.1 mm in diameter (see Figure 1). The entrance part of each die orifice had a taper angle of 9°.

#### 2.2.2. Manufacturing of Thermoplastic Flat Laminates

**Figure 2:**
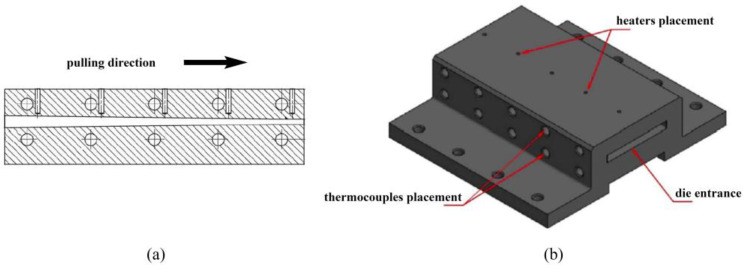
Diagram of the heated die block used to manufacture pultruded thermoplastic flat laminates of 75 mm × 3.5 mm: (**a**) cross section of the heated die block; (**b**) 3D model of the heated die block.

In order to manufacture thermoplastic GF/PP flat laminates of 75 mm × 3.5 mm we used the heated die of 200 mm × 115 mm × 60 mm, and the cooling die of 75 mm × 115 mm × 60 mm, both for commercial PCTs and for inhouse-made PCTs. The heated die block had a 190 mm long tapered section with a tapering angle of 0.72° (see Figure 2).

### 2.3. Morphology Analysis Using an Optical Microscope

In this work we studied the morphology of commercial and inhouse-made PCTs and pultruded GF/PP flat laminates. Specimens of produced profiles were cast in resin and ground with Metprep 3/PH-3 (Allied High Tech Products Inc., Rancho Dominguez, CA, USA). For the final grinding we used the Silicon Carbide Foils FEPA P#2000 (Struers, Ballerup, Denmark). An Axio Scope A1 (Zeiss, Jena, Germany) optical microscope was used for morphology analysis.

### 2.4. Thermal Analysis

Crystallinity of the commercial and inhouse-made PCTs, as well as the pultruded thermoplastic GF/PP flat laminates and bars, was determined in accordance with the technique described in [37] using differential scanning calorimetry (DSC) analysis. The measurements were performed on a DSC-60Plus (Shimadzu, Japan) in a temperature range of 30–200 °C, at a heating rate of 5 °C/min, and an inert gas flow rate of 60 mL/min. To determine the resin weight fraction of the commercial and inhouse-made PCTs as well as the pultruded thermoplastic GF/PP flat laminates and bars we utilized thermogravimetric analysis (TGA). The measurements were performed on a DTG-60 (Shimadzu, Kyoto, Japan), in a temperature range of 30–900 °C, at a heating rate of 5 °C/min, and an argon purge rate of 80 mL/min. The obtained results of the resin weight fraction measurements were then used for the calculation of the fiber volume fraction of the pultruded thermoplastic GF/PP flat laminates and bars.

### 2.5. Mechanical Tests

Flat laminate specimens were machined on a Shtalmark M1-912 M/2 CNC milling machine (Rusintermash Ltd., Pushkino, Russia). Tensile, flexural, and interlaminar shear properties of flat laminate specimens were determined in accordance with ISO 527-2, ASTM D790-15e2, and ASTM D2344 procedures, respectively. Flat laminate specimens were tested on an Instron 5969 testing machine (Instron, Norwood, MA, USA). In the tensile tests we used a clip-on extensometer to measure strain. For the bending modulus measurements we used crosshead displacement with compliance corrections as allowed by paragraph 6.1.5.1 of ASTM D790-15e2. Compliance correction was performed in advance on a rigid (thick metallic) specimen with the same configuration of the load string, in accordance with Appendix X1 of the standard. The Bluehill 3 software (Instron, Norwood, MA, USA) later applied corrections automatically when testing specimens.

To determine the mechanical properties of the pultruded thermoplastic bars we conducted a series of tensile, flexural, and short-beam shear tests in accordance with the ASTM D3039/D3039M, ASTM D4476/D4476M-14, and ASTM D4475-2002 procedures, respectively. Pultruded bars were tested on a DHY-10080 electronic universal testing machine (Hengyi Precision Instrument Co. Ltd., Shanghai, China). In the tensile tests we monitored strain by an extensometer with a gauge length of 25mm on the center surface of the effective test length of the bars. For bending modulus measurements we used crosshead displacement so the specimen did not slip with the loaded crosshead due to the applied fixture.

## 3. Results and Discussions

### 3.1. Fabrication of Inhouse-Made PCTs, Pultruded Thermoplastic Bars, and Flat Laminates

**Figure 3:**
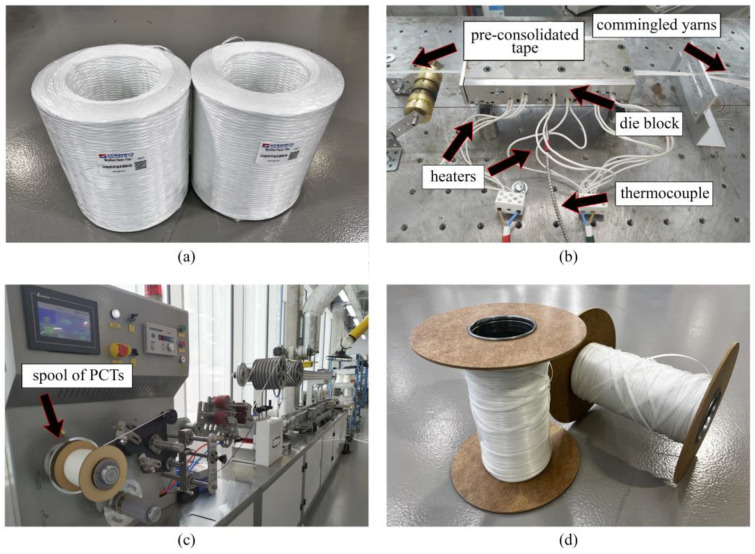
Inhouse fabrication of thermoplastic GF/PP PCTs: (**a**) source material—glass fiber/polypropylene (GF/PP) commingled yarns (Jushi Holdings Inc., USA); (**b**) heated die block with corrugated cavity, used for production of PCTs; (**c**) plastron FLD 35 extrusion machine; (**d**) inhouse-made PCTs.

**Figure 4:**
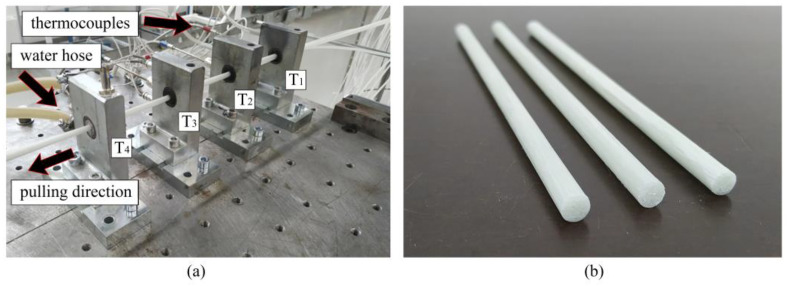
Pultrusion of thermoplastic Ø 6 mm GF/PP bars based on inhouse-made PCTs: (**a**) pultrusion process; (**b**) pultruded bars.

**Figure 5:**
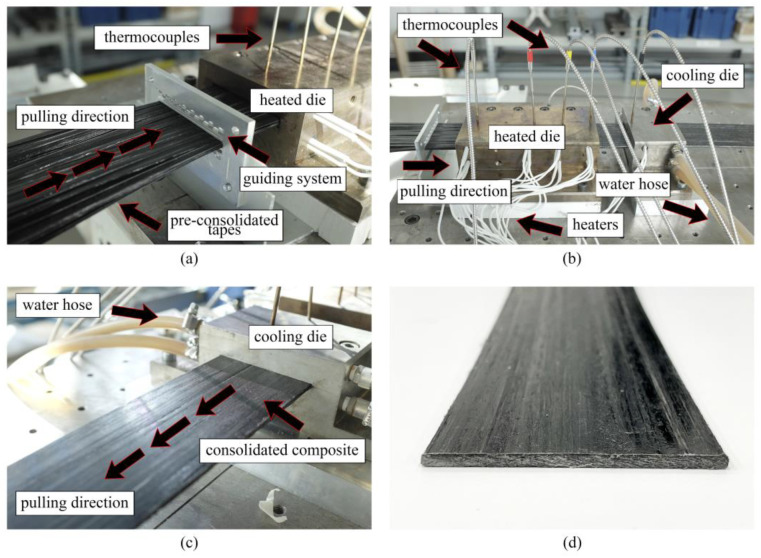
Pultrusion of thermoplastic flat GF/PP laminates based on commercial PCTs: (**a**) commercial PCTs entering the heated die; (**b**) die blocks during the pultrusion; (**c**) consolidated flat laminate leaving the cooling die block; (**d**) pultruded flat laminate.

**Figure 6:**
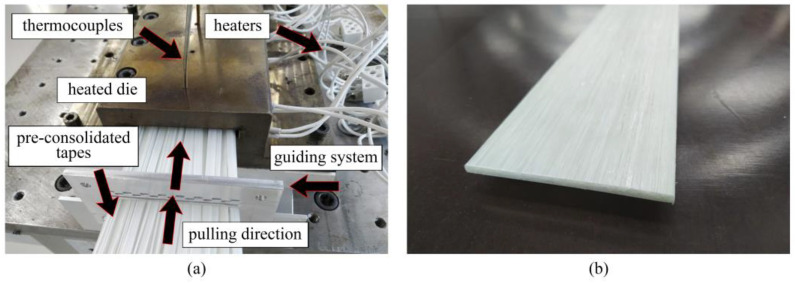
Pultrusion of thermoplastic flat GF/PP laminates based on inhouse-made PCTs: (**a**) inhouse-made PCTs entering the heated die; (**b**) pultruded flat laminate.

Experimental setups used to manufacture inhouse-made PCTs, pultruded thermoplastic bars, and flat laminates are shown in Figure 3, Figure 4, Figure 5 and Figure 6. In order to manufacture inhouse-made PCTs (see Figure 3d) we used four glass fiber/polypropylene (GF/PP) commingled yarns (Jushi Holdings Inc., USA), a modified die block with corrugated cavity walls, and a Plastron FLD 35 extrusion machine presented in Figure 3a–c, respectively. The volume fraction of reinforcement constituted 34.5%. The temperature of the heated die block was set at 240 ± 5 °C and controlled with the help of embedded thermocouples. The pulling speed was 8 m/min. The pre-consolidated tapes had widths of 4.94 mm and thicknesses of 0.55 mm. In total, we produced eighty spools of inhouse-made PCTs with 150 m of tape was wound on each of them (Figure 3d).

To manufacture thermoplastic GF/PP bars with diameters of Ø 6 mm (see Figure 4b) we used eight inhouse-made PCTs. The following temperature conditions were set at the heated dies (Figure 4a)—T_1_ = 200 ± 5 °C, T_2_ = 185 ± 5 °C, T_3_ = 170 ± 5 °C, respectively. To maintain temperature conditions the heaters were installed at the top and bottom of the heated dies. In order to control the temperature, we used thermocouples placed in contact with the heated dies. The temperature of the cooling die was set at T_4_ = 40 ± 10 °C. The pulling speed constituted 0.3 m/min. In total, we produced ten meters of GF/PP bars for further mechanical testing and morphology analysis.

Figure 5 shows the fabrication of flat GF/PP laminates based on commercial PCTs. The laminate had a cross section of 75 mm × 3.5 mm (Figure 5d), and was made of 110 tapes (Figure 5a). At the stationary mode the temperature at the heated die was 200 ± 10 °C and at the cooling die—60 ± 10 °C. In order to control the temperature several thermocouples were embedded into the body of the heated die block (Figure 5b). The pulling speed was set at 0.4 m/min. In total, we produced five meters of flat GF/PP laminates based on commercial PCTs for further mechanical testing and morphology analysis.

Figure 6 shows the pultrusion of flat GF/PP laminates based on inhouse-made PCTs. The laminate had a cross section of 75 mm × 3.5 mm (Figure 6b), and was made of 66 inhouse-made PCTs (Figure 6a). We utilized the same temperature regime as those used for the production of flat GF/PP laminates based on commercial PCTs. The pulling speed was 0.4 m/min. In total, we produced five meters of flat GF/PP laminates based on inhouse-made PCTs for further mechanical testing and morphology analysis.

### 3.2. Results of Morphology Analysis

**Figure 7:**
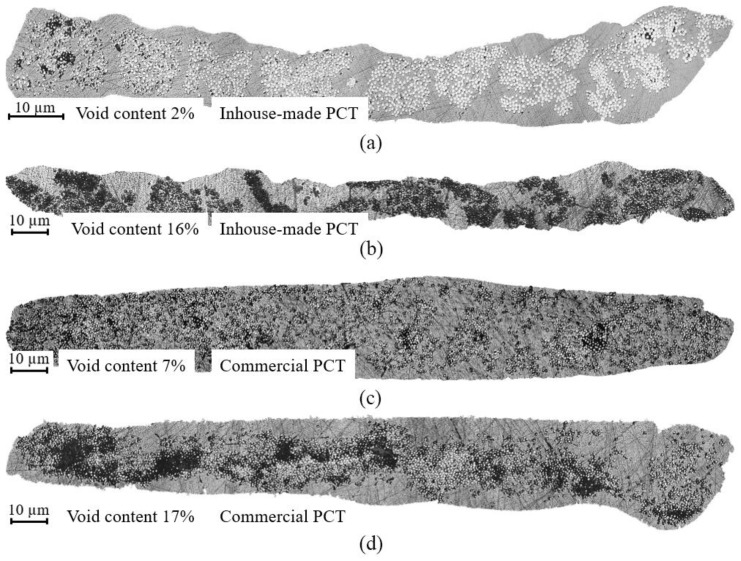
Micrographs of PCTs: (**a**) inhouse-made PCT with the lowest void content of 2%; (**b**) inhouse-made PCT with the largest void content of 16%; (**c**) commercial PCT with the lowest void content of 7%; (**d**) commercial PCT with the largest void content of 17%.

Threshold segmentation was applied to the obtained micrographs of the seven inhouse-made and seven commercial PCTs, and their corresponding void content was calculated. The mean values of the void content were 11% and 14%, respectively. The largest and the lowest values of void content of all the studied inhouse-made PCTs (Figure 7a,b) were 2% and 16%, respectively. The largest and the lowest values of void content of all the studied commercial PCTs (Figure 7c,d) were 7% and 17%, respectively.

In the central part of the inhouse-made PCT with 2% void content (Figure 7a), we could see the proper impregnation of glass fiber reinforcement and a uniform distribution of reinforcing fiber bundles over the whole area of a cross section. Separate pores and un-impregnated fibers could only be seen at the edges of the cross section of the inhouse-made PCTs. Inhouse-made PCTs with 16% void content (see Figure 7b) had resin-rich areas and un-impregnated portions of fiber bundles distributed across the entire cross section.

In commercial PCTs with 7% void content (Figure 7c) we observed a uniform distribution of reinforcing fiber over the whole area of a cross section. However, the fibers were not assembled into fiber bundles. Pores, located between the fiber and the matrix, could be seen across the entire cross section of the tape. Commercial PCTs with 17% void content (Figure 7d) contained pores within the fiber bundles and resin-rich areas.

**Figure 8:**
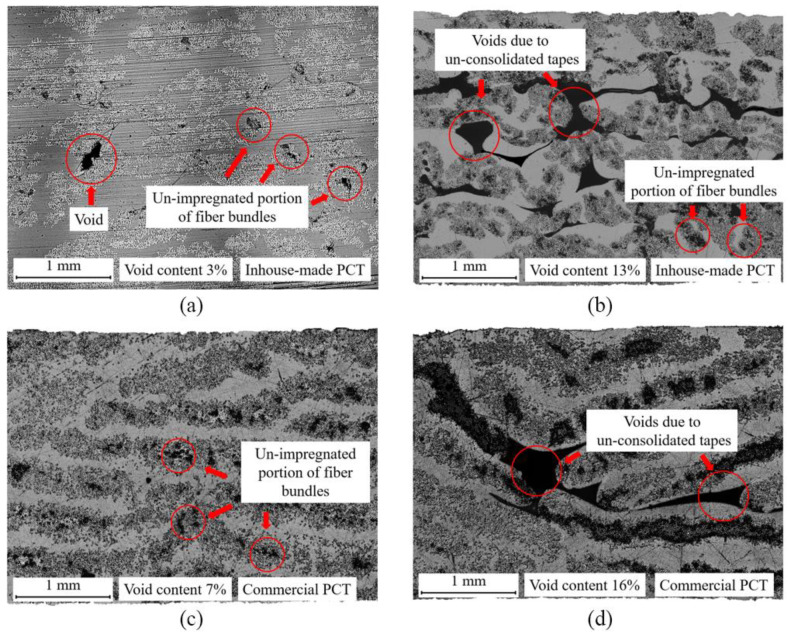
Areas of micrographs of pultruded thermoplastic flat GF/PP laminates: (**a**) flat laminate based on inhouse-made PCTs, area with the lowest void content of 3%; (**b**) flat laminate based on inhouse-made PCT, area with the largest void content of 13%; (**c**) flat laminate based on commercial PCTs, area with the lowest void content of 7%; (**d**) flat laminate based on commercial PCTs, area with the largest void content of 16%.

To calculate void content, threshold segmentation was applied to the obtained micrographs of the pultruded thermoplastic flat GF/PP laminates based on inhouse-made and commercial PCTs. Th mean values of void content were 12% and 15%, respectively. Figure 8 presents two regions with the lowest and highest void content values of laminates based on inhouse-made and commercial PCTs.

The flat laminates based on inhouse-made PCTs (Figure 8a,b) contained voids that had arisen on the border of the un-consolidated tapes in the center of the profile, which was caused by insufficient heating of the material during pultrusion. The flat laminates contained un-impregnated portions of fiber bundles, the presence of which was previously observed in the inhouse-made PCTs.

The flat laminates based on commercial PCTs (Figure 8c,d) featured un-impregnated regions within the fiber bundles, together with resin-rich areas and voids between un-consolidated tapes.

According to the results of TGA analysis and further calculations, the fiber volume fractions of pultruded GF/PP bars based on inhouse-made PCTs and flat GF/PP laminates based on commercial and inhouse-made PCTs were 34.1%, 32.0%, and 36.0%, respectively.

### 3.3. Results of Thermal Analysis

The results of DSC, TGA analysis, and the calculated crystallinity values of commercial and inhouse-made PCTs, pultruded thermoplastic GF/PP, and flat laminates and bars are presented in Table 1. The crystallinity of each material was obtained as follows [37]:

(1) $$X_{c}=\frac{1}{w}\frac{\Delta H_{m}}{\Delta H_{m}^{0}}\times 100\%$$

where w is the resin mass fraction, $\Delta H_{m}$ is the enthalpy for melting, $\Delta H_{m}^{0}$ is the enthalpy of melting for a 100% crystalline polypropylene sample adopted from [38].

The crystallinity of inhouse-made PCTs was higher than those of the commercial PCTs. As a result, the crystallinity of thermoplastic pultruded composites based on inhouse-made PCTs was also higher than their counterparts made of commercial PCTs. The manufacturing of inhouse-made PCTs and pultrusion of thermoplastic composites was performed at the laboratory under the same normal conditions. However, the crystallinity of thermoplastic composites was higher compared to the PCTs due to the difference in the cooling rate caused by the larger geometrical dimensions of the composite profiles. The cooling rate of GF/PP flat laminates in the cooling die was around 7 K/s, while the cooling rate of PCT after the die exit was around 10 K/s.

### 3.4. Mechanical Testing Results

**Figure 9:**
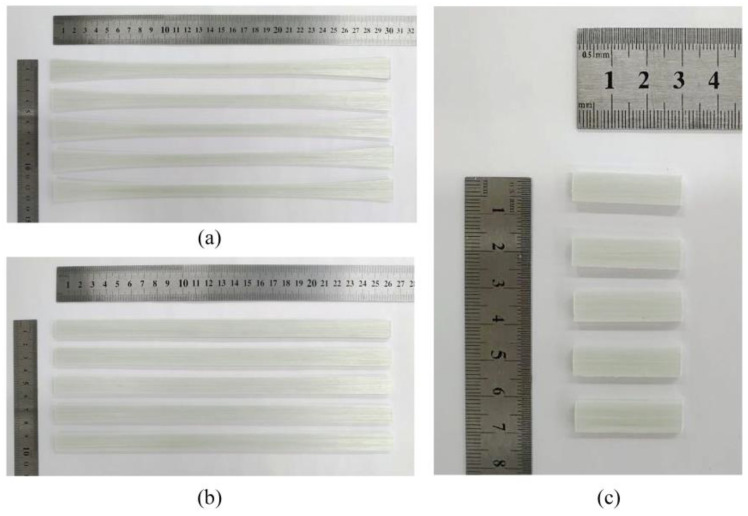
Mechanical test specimens machined from pultruded GF/PP flat laminates based on inhouse-made PCTs: (**a**) tension test specimens; (**b**) flexure test specimens; (**c**) short-beam shear test specimens.

**Figure 10:**
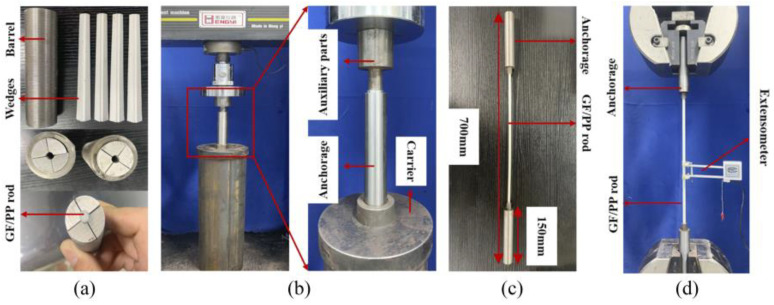
Test setup for tension testing of pultruded thermoplastic Ø 6mm GF/PP bars based on inhouse made PCTs: (**a**) anchorage design and assembly; (**b**) pre-tightening anchor; (**c**) anchored specimen; (**d**) tensile testing.

**Figure 11:**
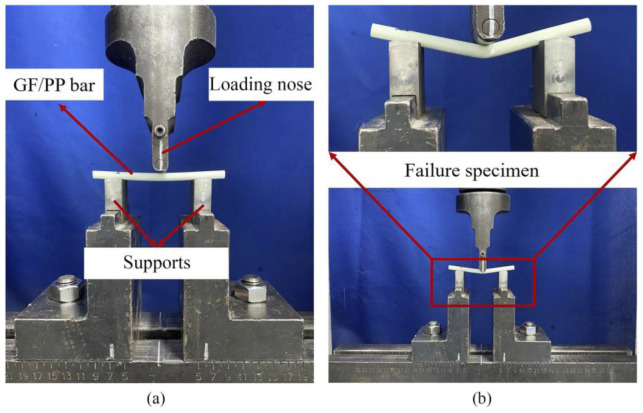
Test setup for flexural testing of pultruded thermoplastic Ø 6mm GF/PP bars based on inhouse made PCTs: (**a**) flexural testing; (**b**) failure specimen.

**Figure 12:**
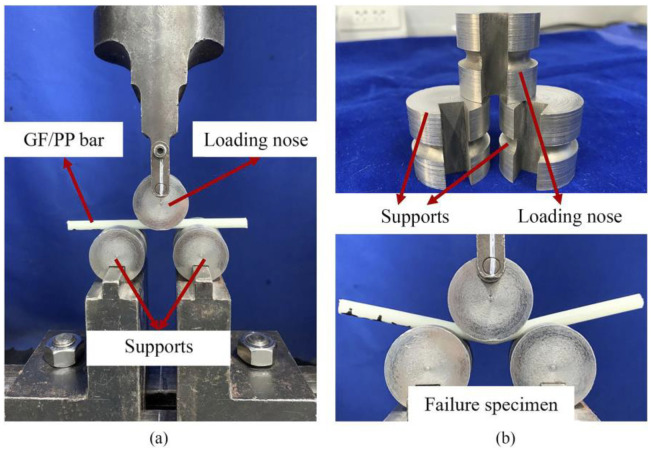
Test setup for short-beam shear testing of pultruded thermoplastic Ø 6 mm GF/PP bars based on inhouse-made PCTs: (**a**) short-beam shear testing; (**b**) failure specimen.

Specimens of 75 mm × 3.5 mm flat GF/PP laminates prepared for flexural and short-beam shear tests had dimensions of 260 mm × 13 mm × 3.5 mm (Figure 9b) and 30 mm × 8 mm × 3.5 mm (Figure 9c), respectively. The shape of specimens for tensile tests was non-standard (Figure 9a) and were adopted as in [22]. Specimens of GF/PP bars Ø 6 mm prepared for tensile, flexural, and short-beam shear tests had lengths of 700 mm, 80 mm, and 100 mm, respectively. Five specimens were prepared for each type of test. The test setups for tension, flexure, and short-beam shear tests of pultruded thermoplastic GF/PP bars Ø 6 mm are shown in Figure 10, Figure 11 and Figure 12, respectively.

**Figure 13:**
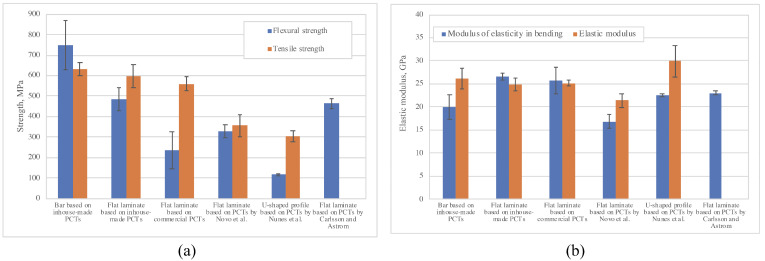
Mechanical properties of pultruded thermoplastic composites: (**a**) flexural and tensile strength; (**b**) modulus of elasticity in bending and elastic modulus.

Results of mechanical testing of pultruded thermoplastic GF/PP bars of Ø 6 mm and of 75 mm × 3.5 mm flat laminates are shown in Table 2 and Figure 13. The laminates produced from inhouse-made PCTs had higher flexural, tensile, and apparent interlaminar shear strength compared to those made from commercial PCTs, by as much as 106%, 6.4%, and 27.6%, respectively. The lower quality of commercial PCTs as compared to the inhouse-made ones resulted in an inferior mechanical performance of the corresponding pultruded laminates. The injection method used for the production of commercial PCTs did not allow the uniform impregnation of glass fiber reinforcement by polypropylene resin. This resulted in the formation of un-impregnated regions and matrix cracks within the core region of fiber bundles in commercial PCTs. These defects, in turn, resulted in debondings and branched matrix cracks in the flat laminates based on commercial PCTs. On the contrary, the use of commingled yarns consisting of matrix and glass fibers properly intermingled over the whole length of the “prepreg” material making it possible to achieve a more uniform impregnation of the inhouse-made PCTs and, therefore, to produce higher quality pultruded profiles.

For comparison purposes, the values of the mechanical properties of the pultruded thermoplastic composites manufactured from PCTs described in the previous studies [22,23,26] are given in Table 2 in italics. The proposed method of producing PCTs based on commingled yarns made it possible to obtain pultruded composite laminates with better mechanical properties and with larger cross section dimensions than their counterparts described in the literature. For example, the thermoplastic composites manufactured in the course of this study exhibited the highest mechanical characteristics in flexure (such as flexural strength, modulus of elasticity in bending, flexural strength/volume fraction, and modulus of elasticity in bending/volume fraction) and tension (such as tensile strength, tensile strength/volume fraction, and elastic modulus/volume fraction) as compared to their analogues studied previously (Table 2).

## 4. Conclusions

The study investigated the influence of the PCT manufacturing method on the mechanical properties and morphology of pultruded thermoplastic composites and proposed a method for producing PCTs based on the use of commingled yarns. Two types of pultruded thermoplastic GF/PP profiles were produced in the course of the study—Ø 3.5 mm bars and 75 mm × 3.5 mm flat laminates. In order to study the mechanical properties of the produced composites, we conducted a series of tensile, flexural, and short-beam shear tests. We also studied the morphology of both of the inhouse-made and commercial PCTs and of pultruded composites based thereupon.

Results showed that flat laminates produced from inhouse-made PCTs had better mechanical properties than those manufactured from commercial PCTs. This could be explained by a better impregnation of glass fiber reinforcement in the inhouse-made PCTs produced from commingled yarns, as shown by optical microscopy. On the contrary, the injection method could not provide uniform impregnation, which resulted in the formation of un-impregnated regions and matrix cracks over the whole cross section of the commercial PCTs, thus impairing the mechanical performance of corresponding pultruded composites. Defects formed during the manufacture of commercial PCTs coul not be corrected during pultrusion and led to the formation of debondings and branched matrix cracks in the pultruded composites. The comparison of mechanical characteristics and cross section dimensions of pultruded thermoplastic composites described earlier in the literature and those produced from inhouse-made PCTs demonstrated the great potential of the proposed method of PCT manufacturing.

In further studies, the authors intend to investigate the influence of PCT quality on the durability of corresponding pultruded thermoplastic profiles subjected to water, seawater, and alkaline environments. Moreover, the effects of technological regimes of PCT manufacturing and thermoplastic pultrusion on mechanical properties, occurrence of manufacturing-induced shape distortions, porosity, and morphology of corresponding composites will be studied as well. In particular, the authors plan to investigate the influence of pulling speed [39,40], temperature regime, presence of additives [41], and carbon nanotubes [42]. The obtained experimental results will be used for the further multicriteria optimization of pultrusion manufacturing conditions [43,44].

## Figures and Tables

**Table 1:** Results of thermal analysis.

	$\Delta H_{m}^{0}[J/g]$	$\Delta H_{m}[J/g]$	w[%]	$X_{c}[\%]$
Inhouse-made PCTs	207	22.5	39.7	27.3
Commercial PCTs		17.6	37.1	22.9
Bar based on inhouse-made PCTs		23.1	35.7	31.2
Flat laminate based on inhouse-made PCTs		22.2	33.7	31.8
Flat laminate based on commercial PCTs		17.7	36.8	23.3

**Table 2:** Mechanical properties of pultruded thermoplastic composites.

	Bar Based on Inhouse-Made PCTs	Flat Laminate Based on Inhouse-Made PCTs	Flat Laminate Based on Commercial PCTs	*Flat Laminate Based on PCTs by Novo* et al. [22]	*U-Shaped Profile Based on PCTs by Nunes* et al. [23]	*Flat Laminate Based on PCTs by Carlsson and Astrom* [26]
Fiber volume fraction [-]	0.34	0.36	0.32	*0.30*	*0.56*	*0.35*
Flexural strength [MPa]	750 ± 118 CV = 15.7%	485 ± 58 CV = 11.9%	235 ± 91 CV = 38.8%	*329 ± 30* *CV = 9.1%*	*117 ± 4* *CV = 3.7%*	*465 ± 24* *CV = 5.2%*
Modulus of elasticity in bending [GPa]	20.0 ± 2.7 CV = 13.4%	26.6 ± 0.8 CV = 2.9%	25.7 ± 2.9 CV = 11.3%	*16.8 ± 1.5* *CV = 8.9%*	*22.5 ± 0.3* *CV = 1.3%*	*23 ± 0.45* *CV = 2%*
Flexural strength/Fiber volume fraction [MPa]	2206 ± 347	1347 ± 160	734 ± 285	*1097 ± 100*	*209 ± 8*	*1329 ± 69*
Modulus of elasticity in bending/Fiber volume fraction [GPa]	58.7 ± 7.9	73.9 ± 2.1	80.3 ± 9.0	*56 ± 5*	*40.2 ± 0.5*	*65.7 ± 1.3*
Tensile Strength [MPa]	632 ± 31 CV = 5.0%	597 ± 55 CV = 9.2%	561 ± 35 CV = 6.2%	*356 ± 53* *CV = 15.0%*	*305 ± 26* *CV = 8.5%*	*-*
Elastic modulus [GPa]	26.2 ± 2.2 CV = 8.4%	24.9 ± 1.4 CV = 5.6%	25.2 ± 0.7 CV = 2.8%	*21.4 ± 1.5* *CV = 7%*	*29.9 ± 3.5* *CV = 11.7%*	*-*
Tensile Strength/Fiber volume fraction [MPa]	1859 ± 92	1658 ± 153	1753 ± 109	*1186 ± 177*	*545 ± 46*	*-*
Elastic modulus/Fiber volume fraction [GPa]	77.1 ± 6.4	69.2 ± 3.9	78.8 ± 2.2	*71.3 ± 5.0*	*53.4 ± 6.3*	*-*
Apparent interlaminar shear strength [MPa]	19.3 ± 0.5 CV = 2.56%	23.1 ± 1.6 CV = 6.87%	18.1 ± 3.4 CV = 18.8%	*-*	*-*	*-*
Cross-section of a composite profile and its dimension [mm]	bar Ø 6	flat laminate 75 × 3.5	flat laminate 75 × 3.5	*flat laminate* *20 × 3*	*U-shaped profile* *24 × 4 × 2*	*flat laminate* *30 × 3*

## Data Availability

The data presented in this study are available on request from the corresponding author.
